# Targeted Drug Delivery via the Use of ECM-Mimetic Materials

**DOI:** 10.3389/fbioe.2020.00069

**Published:** 2020-02-18

**Authors:** Jeongmin Hwang, Millicent O. Sullivan, Kristi L. Kiick

**Affiliations:** ^1^Department of Biomedical Engineering, University of Delaware, Newark, DE, United States; ^2^Department of Chemical and Biomolecular Engineering, University of Delaware, Newark, DE, United States; ^3^Department of Materials Science and Engineering, University of Delaware, Newark, DE, United States

**Keywords:** targeted drug delivery, extracellular matrix, hydrogel, ECM ligand, ECM cell receptors

## Abstract

The use of drug delivery vehicles to improve the efficacy of drugs and to target their action at effective concentrations over desired periods of time has been an active topic of research and clinical investigation for decades. Both synthetic and natural drug delivery materials have facilitated locally controlled as well as targeted drug delivery. Extracellular matrix (ECM) molecules have generated widespread interest as drug delivery materials owing to the various biological functions of ECM. Hydrogels created using ECM molecules can provide not only biochemical and structural support to cells, but also spatial and temporal control over the release of therapeutic agents, including small molecules, biomacromolecules, and cells. In addition, the modification of drug delivery carriers with ECM fragments used as cell-binding ligands has facilitated cell-targeted delivery and improved the therapeutic efficiency of drugs through interaction with highly expressed cellular receptors for ECM. The combination of ECM-derived hydrogels and ECM-derived ligand approaches shows synergistic effects, leading to a great promise for the delivery of intracellular drugs, which require specific endocytic pathways for maximal effectiveness. In this review, we provide an overview of cellular receptors that interact with ECM molecules and discuss examples of selected ECM components that have been applied for drug delivery in both local and systemic platforms. Finally, we highlight the potential impacts of utilizing the interaction between ECM components and cellular receptors for intracellular delivery, particularly in tissue regeneration applications.

## Introduction

Conventional drugs have been critical to the effective management of disease. Despite the benefits of free drugs, 118 drugs approved from 1980 to 2009 in the United States were withdrawn from the market, 22% of them due to safety reasons including hepatic toxicity, severe cardiovascular effects, gastrointestinal issues, and allergic reactions (Qureshi et al., 2011; Prasad, 2014). In addition, safety concerns and inadequate efficacy (78%) were the main reasons for the failure of 54% of the 640 therapeutics that entered phase 3 trials between 1998 and 2008, with follow-up through 2015 (Hwang et al., 2016). The pharmacokinetics of any drug compound, including its efficacy and safety, is critically affected by the route of drug entry (Tibbitt et al., 2016). For example, systemically administrated drugs are exposed to the entire circulatory system, and may access multiple tissues/organs within the body in the absence of direct targeting (Blanco et al., 2015); for drugs with intracellular targets, additional challenges are posed by the need to navigate the intracellular landscape. The challenges are compounded for drugs that are highly toxic to healthy cells, such as cytostatic drugs for chemotherapeutics or immunosuppressants, adding an extra set of barriers during pre-clinical and clinical evaluation.

To overcome the pharmacokinetic limitations of free drugs, drug delivery systems (DDS) have been designed based on nanomaterials, polymers, and lipids, which can be attached to drugs or used to encapsulate drugs in order to better localize their delivery or control drug release over extended periods (Langer, 1998; Hu et al., 2016; Kanamala et al., 2016; Liu et al., 2016). Nanometer-scale therapeutics can extravasate from circulation and accumulate in some tissues via passive targeting effects (Allen and Cullis, 2004). Such advances were the basis of the improvements in chemotherapy efficacy using liposomal formulations of doxorubicin (Doxil), which was introduced for the treatment of Kaposi’s sarcoma in 1995. In addition, over 340 DDS have been approved by the FDA and employed clinically to date (Table 3 from Zhong et al., 2018), and it is clear that nanomaterial DDS have great potential for the targeted delivery of drugs. However, passive targeting is only useful for targeting very specific organs such as tumors (Torchilin, 2014), and even in those cases, some regions of tumors exhibit variations in microvascular permeability that diminish the efficacy of passive targeting.

Local administration provides a simple strategy to enhance active targeting to specific sites, taking advantage of physical localization (Panyam and Labhasetwar, 2003). Employing DDS for localized therapy can improve drug efficacy by preventing the loss of therapeutic agent from the administration site, which minimizes necessary doses and maximizes potency. In addition, polymeric or liposomal carriers can be tailored to achieve sustained release of drugs at optimal therapeutic concentrations in a particular tissue (Sheikhpour et al., 2017; Cervadoro et al., 2018; Raave et al., 2018). As DDS for localized therapy, hydrophilic polymeric hydrogels (for hydrophilic drugs) or nanoparticles (for encapsulation of hydrophobic drugs) can be directly injected or applied to the tissue of interest to achieve sustained and controlled drug release to a particular site through diffusion (Kohane and Todd, 2008; Tibbitt et al., 2016). The tailoring of hydrogel and nanoparticle composition, structure, and porosity has been possible owing to the enormous range of polymers and crosslinking chemistries developed for these applications.

Hydrogels have been designed to exploit the mechanical and biochemical activities of the native extracellular matrix (ECM) to influence cells through cell-matrix interactions (Kharkar et al., 2013; Cai and Heilshorn, 2014; Caliari and Burdick, 2016; Ooi et al., 2017; Zhang and Khademhosseini, 2017). These cell-matrix interactions are pivotal to enhance cell infiltration into the hydrogel and promote cell responses in hydrogels that are appropriate for tissue regeneration and drug delivery applications. To create hydrogels that support cell-matrix interactions, ECM molecules are often utilized in hydrogel formulations. For example, decellularized ECM (dECM) matrices derived from tissues and organs are composed of native ECM molecules, and dECM therefore mimics the structural properties of the native matrix (Crapo et al., 2011; Saldin et al., 2017). Owing to the preservation of biochemical cues from the native tissue microenvironment, dECM matrices trigger cellular response that have been exploited clinically in tissue engineering and regenerative medicine [Tissue Mend^®^ (Stryker Orthopaedics, United States), AlloDerm^®^ (LifeCell Corp. United States), CutffPatch^TM^ (Organogenesis, United States)]. In addition, the delivery of growth factors (Seif-Naraghi et al., 2012) and microRNA (Hernandez et al., 2018) using dECM has recently been explored.

Owing to the myriad cellular interactions with ECM-based materials, the surfaces of drug-loaded nanoparticles also have been modified with ECM-based materials to increase the extent of ligand-mediated, site-specific DDS. The incorporation of bio-specific ligands such as proteins, polysaccharides, peptides, aptamers, and small molecules, facilitates interaction with specific receptors that are either over-expressed or expressed only in specific tissues or cells to achieve active targeting. For example, it has been reported that α_v_β_3_ integrin and CD44 receptors are upregulated in various tumor tissues (Danhier et al., 2010). The RGD sequence derived from multiple ECM proteins to target integrin receptors, and hyaluronic acid to target CD44 receptor on cancerous cells, have been widely employed to transport anti-tumor agents (Murphy et al., 2008; Danhier et al., 2012; Huang and Huang, 2018; Fu et al., 2019). Furthermore, target receptor-mediated siRNA delivery has been developed utilizing ligands such as peptides, GalNAc, and aptamers (Nikam and Gore, 2018). Alnylam Pharmaceuticals launched the first RNA interference (RNAi) drug, ONPATTRO^®^, which uses lipid nanoparticles to deliver RNAi intravenously and treat polyneuropathy caused by hereditary ATTR amyloidosis (Garber, 2018). As next-generation alternatives of ONPATTRO^®^, the GalNac ligand has been employed to target asialoglycoprotein receptor (ASGP-R) on the hepatocytes. ASGP-R has been shown to mediate endocytosis and degradation of wide variety of desialylated glycoproteins and neoglycoproteins which contain GalNAc residues on the their N-linked carbohydrate chains, and it recognizes specific markers of autoimmune hepatitis (Roggenbuck et al., 2012). The GalNAc conjugated RNAi systems for treatment of liver diseases are currently in phase III (Table 1 from Morrison, 2018). Thus, active targeting strategies have great potential to optimize the delivery of intracellularly active drugs such as many small molecules, as well as biomacromolecules including nucleic acids, peptides, or proteins, which require specific endocytic pathways for action.

Here, we focus on recent developments in the use of ECM components for actively targeted DDS. In particular, we briefly review ligand-receptor mediated endocytosis and cellular interactions with various ECM components as targeting strategies, and we consider the advantages afforded by each approach. We then provide examples of the use of key ECM components in DDS, either as hydrogels or as ligands applied for targeted intracellular DDS.

## Ecm-Cell Interaction Mediated Drug Delivery Applications

Researchers have exploited an expanded understanding of the interactions between cells and the ECM, as well as increased knowledge about signaling pathways and molecules relevant to the treatment of disease, in designing new, more cell-specific therapeutics and DDS. Cell surface receptors are attractive pharmacological targets since they transduce signals from the extracellular environment to modulate cell responses. Integrins, a major class of transmembrane receptors whose primary role is to recognize and bind ECM, have been a target of therapeutic development for nearly 30 years in the pharmaceutical industry (Goodman and Picard, 2012; Raab-Westphal et al., 2017). However, despite some promising therapeutic advances, the complex biology of integrins has often confounded drug development. Integrins are involved in canonical processes ranging from embryonic development to mature tissue function through binding to their ligands. Therefore, it is critical to understand the mechanisms by which cell-ECM interactions enable cells to sense and respond to extracellular signals encoded in the matrix.

**FIGURE 1:**
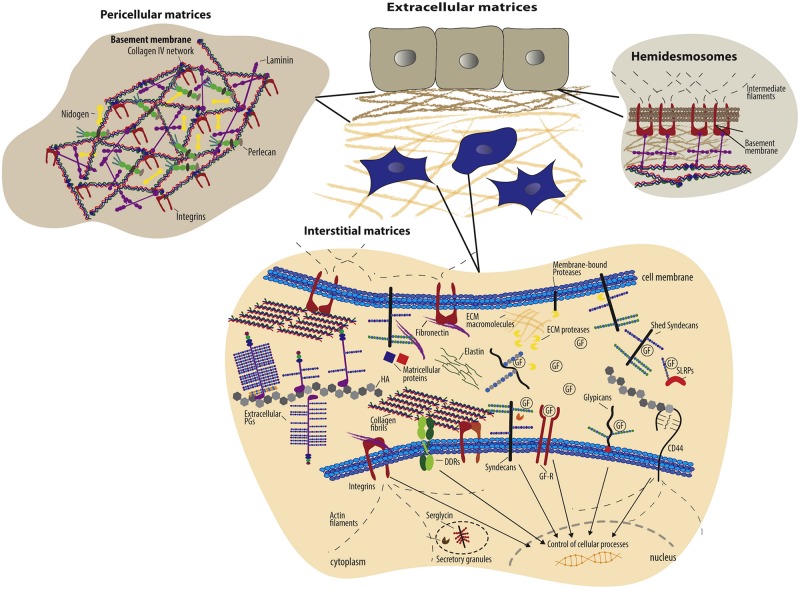
Schematic overview of extracellular matrix components and their cell surface receptors (Theocharis et al., 2016). Copyright 2016. Reproduced with permission from Elsevier Inc. Cells have specific surface receptors, such as integrins, cell surface proteoglycans (ex. syndecans and glypicans), the HA receptor CD44, and DDRs, to bind ECM components for regulation of various cellular functions.

Each ECM molecule has an affinity to a cell surface receptor or receptors, including integrins (Figure 1); moreover, the specific integrins expressed by a given cell depend both on the cell type as well as on the cell’s physiological state. Accordingly, DDS can be modified with ECM molecules to serve as ligands that will facilitate drug targeting. These approaches are described below for various classes of ECM that have been particularly fruitful in targeted delivery.

### Types of ECM Molecules

#### Proteins

Extracellular matrix proteins include fibrous proteins such as collagen and elastin, and glycoproteins such as fibronectin, laminins, vitronectin, thrombospondin, chondronectin, osteonectin, and fibrin. Collagen is a major ECM component that provides mechanical support, regulates cellular behavior, and directs tissue development. Collagen fibrils, which are formed by self-assembly of triple helical collagen molecules, are cross-linked to provide mechanical strength and integrity to the ECM, and collagens strongly influence the tensile strength and elasticity of tissue. In addition, collagens interact with integrins to regulate cell adhesion, proliferation, and migration, and collagens also interact with other ECM components to direct matrix remodeling (Leitinger and Hohenester, 2007). Fibronectin also regulates a wide variety of cellular functions including cell adhesion, migration, growth and proliferation, embryonic morphogenesis, and wound healing (Pankov and Yamada, 2002; Zollinger and Smith, 2017). Fibronectin usually exists as a dimer composed two nearly identical subunits (type I, type II, and type III) linked together through disulfide bond formation at their C-termini. The type III subunit contains about 100 amino acids in two anti-parallel β-sheets, which are also present in collagens, and the type III subunit also encodes integrin binding (via the RGD motif) and heparin-binding domains. Laminins promote cell adhesion and migration, neurite outgrowth, angiogenesis. Laminins are a major component of basement membrane along with collagen type IV, with a structure that is comprised of heterotrimeric glycoproteins; three subunits, α, β, and γ, come together to form at least 19 laminin isoforms (Colognato and Yurchenco, 2000; Yao, 2017). These laminin isoforms are specifically expressed in tissues to promote biological activities, including cell differentiation, cell shape and movement, and managing tissue phenotypes and survival. The isoforms can bind to other laminins, proteoglycans, and other ECM proteins via various integrins receptors. Due to the ability of ECM proteins to influence cell fate via interactions with integrins, the biocompatible and biodegradable ECM proteins are widely used natural materials for biomedical application (Ramshaw et al., 2009; Benton et al., 2014; Hinderer et al., 2016).

#### Polysaccharides

Extracellular matrix polysaccharides including heparin sulfate, chondroitin sulfate, dermatan sulfate, keratin sulfate and hyaluronic acid provide largely a structural network, as most ECM polysaccharides are not directly involved in cellular interactions, but indirectly through interaction with other proteins. Heparan sulfate/heparin is a linear polysaccharide of repeating N-acetyl glucosamine (GlcNAc)-D-glucuronic acid (GlcA) disaccharide units (Meneghetti et al., 2015), and is often covalently attached to cell-associated proteins such as the syndecans (SDCs) and glypicans (GPCs) to form heparan sulfate proteoglycans (HSPGs) (Christianson and Belting, 2014). HSPGs such as syndecans and glypicans are able to modulate the cellular uptake of bound ligands; in addition, heparin interacts with various proteins to regulate biological process including growth factor or cytokine signaling, coagulation factor activity, microbe-host interactions, and lipoprotein metabolism (Belting, 2003). The interaction is highly specific, involving electrostatic forces between the negatively charged heparin and positively charged amino acid residues (e.g., lysine and arginine), and both protects the stability of proteins and increases their affinity for cell receptors (Gospodarowicz and Cheng, 1986). Due to the ability of heparin to interact with proteins, particularly growth factors, heparin has been utilized widely in DDS, with a focus on binding of growth factors (rather than to cell-surface receptors). Heparin-based hydrogels have been widely employed as growth factor carriers for tissue regeneration (Zieris et al., 2010, 2011; Prokoph et al., 2012; Tsurkan et al., 2013; Liang and Kiick, 2014; Freudenberg et al., 2016).

On the other hand, chondroitin sulfate and hyaluronic acid have an affinity to non-integrin cell receptors such as CD44. Chondroitin sulfate is also composed of a sulfated β-1,3-linked N-acetyl galactosamine (GalNAc) and β-1,4-linked D-glucuronic acid (GlcA) disaccharide repeating units. The sulfation pattern defines the different roles of chondroitin sulfate and its selective interaction with molecules mediating such functions as regulation of signal transduction, cell division and morphogenesis, and development of the central nervous system (Zhao et al., 2015). Hyaluronic acid is a non-sulfated polysaccharide composed of disaccharide repeating units of glucuronic acid and N-acetylglucosamine. Hyaluronic acid as a major role in tissue architecture, tissue regeneration, ingrowth of blood vessels, and cellular functions such as motility, adhesion, and proliferation (Jiang et al., 2011) has been utilized in DDS to improve long-acting and target-specific delivery (Tripodo et al., 2015; Highley et al., 2016; Jiao et al., 2016). In particular, due to the highly specific cellular receptor interaction and cellular uptake of hyaluronic acid in kidney, liver, lymphatic vessels, and tumor sites, hyaluronic acid often has been employed as carriers for intracellular drugs such as anti-tumor agents, and nucleic acids (Oh et al., 2010; Dosio et al., 2016; Lallana et al., 2017; Huang and Huang, 2018; Miyazaki et al., 2018).

### Interactions of ECM With Cell Receptors

Extracellular matrix molecules typically interact with cells through both integrin and non-integrin cell surface receptors (Table 1). The integrin receptors primarily bind the ECM proteins to connect with the cytoskeleton and to cooperate with growth factor receptors for cell survival, cell cycle progression, and cell migration (Giancotti and Ruoslahti, 1999; Giancotti, 2003; Harburger and Calderwood, 2009). As introduced above, integrins consist of heterodimeric non-covalent association of α and β subunits which comprise a specific receptor. In particular, α subunits have a highly specific role in ligand binding for signal transduction (Rosso et al., 2004), with α_2_β_1_, for example, binding to the collagen family, α_5_β_1_ binding to fibronectin, and α_v_β_3_ binding to fibronectin, vitronectin and fibrinogen as summarized in Table 1 from Alam et al. (2007). Integrin-mediated binding has been leveraged for an enormous range of applications, as multiple integrin receptors, including α_v_β_3_, α_v_β_5_, α_v_β_6_, α_v_β_8_, α_IIb_β_3_, α_5_β_1_, and α_8_β_1_ recognize and bind to the Arg-Gly-Asp (RGD) motif which is found in multiple ECM proteins including collagens, fibronectin, laminin, tenascin, vitronectin, and thrombospondin (Ruoslahti and Pierschbacher, 1986; Kim et al., 2011). The RGD sequence as a “minimal” ligand for multiple integrins has been widely used over numerous decades in the development of targeted polymeric and nanoparticle-based therapies. The selectivity of RGD peptide for a specific integrin can be modulated by conformation of the RGD sequence and its flanking residues (Dunehoo et al., 2006). Cyclic peptides, cRGDfK, cRGDyK, and RGDC4 are selective for the integrins α_v_β_3_ and α_v_β_5_, which are overexpressed in vasculature of tumor tissue. Likewise, the GFOGER sequence of collagen binds to four different integrin cell receptors (α_1_β_1_, α_2_β_1_, α_10_β_1_, and α_11_β_1_) (Zeltz et al., 2014); since the α_2_β_1_ integrin receptor is involved in osteogenesis, the GFOGER sequence has been utilized to assist in bone repair (Wojtowicz et al., 2010).

**TABLE 1:** The extracellular matrix components and their cell surface receptors.

	**Integrin**	**Non-integrin receptors**
Collagen	α_1_β_1_, α_2_β_1_, α_10_β_1_, α_11_β_1_	Discoidin domain receptors (DDR1 and DDR2), GPVI (platelets), LAIR (immune cell), OSCAR (osteoblast), and mannose receptors (Endo180 or uPARAP), syndecan, CD44
Fibronectin	α_5_β_1_, α_3_β_1_, α_8_β_1_, and α_v_β_3_, α_4_β_1_, α_4_β_7_, α_9_β_1_,	Syndecan
Laminin	α_1_β_1_, α_2_β_1_, α_3_β_1_, α_6_β_1_, α_7_β_1_, α_10_β_1_, α_6_β_4_, α_v_β_8_	Syndecan, α-dystroglycan CD44
Heparan sulfate		Syndecan, glypicans
Chondroitin sulfate		CD44, NG2, RPTP-σ, GPI-brevican
Hyaluronic acid		CD44, RHAMM, Toll-like receptors

The REDV sequence from fibronectin is a cell adhesion motif to integrin α_4_β_1_, selective for the endothelial cells (Mould et al., 1991; Massia and Hubbell, 1992). Owing to the specificity toward endothelial cells, the REDV sequence has been modified on the system to transport gene to vascular endothelial cells (Wang et al., 2015; Zhou et al., 2016) In addition, the active peptide sequences from laminin are able to interact with integrins, syndecans, α-dystroglycan, and CD44, to perform various biological activities, cell adhesion and neurite outgrowth and proliferation, and angiogenesis, such as those mediated by laminin (Farrukh et al., 2017). The YIGSR sequence and IKVAV sequence from laminin are also cell adhesion domains (Graf et al., 1987; Tashiro et al., 1989), and the RKRLQVQLSIRT (AG73) sequence derived from the mouse laminin α1 chain interacts with syndecans to promote cell adhesion, neurite outgrowth, and angiogenesis (Hoffman et al., 2001). In contrast, DFKLFAVYIKYR-GGC (C16Y), derived from the mouse laminin γ1 chain, binds to integrin α_v_β_3_ and α_5_β_1_ receptors (Hamano et al., 2012). Laminin-derived peptides have been incorporated into the delivery systems of anti-tumor agents to enhance their specificity to highly expressed laminin receptors on cancer cells, including YIGSR for the 32/67 kD receptor, IKVAV for the α_3_β_1_ and α_6_β_1_ integrin receptors, AG73 for syndecan-2 receptor and C16Y for the α_v_β_3_ integrin receptors (Dubey et al., 2010; Negishi et al., 2011; Hamano et al., 2012; Okur et al., 2016; Negishi and Nomizu, 2019).

Short synthetic peptides derived from ECM proteins retain the integrin-binding function, thus are attractive in the design of materials. For example, the Stupp group has developed bioactive peptide amphiphiles (PA) for regenerative medicine applications (Boekhoven and Stupp, 2014; Hendricks et al., 2017; Sato et al., 2018). The RGDS sequence has been attached to PA to induce integrin-mediated adhesion, spreading or migration of fibroblasts, breast cancer cells, and bone marrow mononuclear cells *in vitro* (Storrie et al., 2007; Webber et al., 2010; Zhou et al., 2019). In addition, the IKVAV sequence has been added to PA to induce differentiation of progenitor cells into neurons (Silva et al., 2004). In addition, these ECM proteins have binding sites for both integrin and growth factors. Once ECM proteins engage integrins for adhesion, the proximity of the cell to the ECM localizes the growth factors to their cell surface receptors to induce and/or amplify the signaling for development or repair. Capitalizing on this biological cooperativity offers an enormous advantage in ECM protein-based systems for delivery of growth factors, particularly, in inflammatory diseases where the growth factors are easily degraded (Park et al., 2017). ECM protein-based DDS are able to protect growth factors while delivering them to their receptor sites to regulate cellular responses.

Non-integrin cell receptors for ECM molecules include CD36, certain laminin-binding proteins, and proteoglycans (Rosso et al., 2004) comprising glycosaminoglycan (GAG) chains such as heparan sulfate, chondroitin sulfate, dermatan sulfate and keratin sulfate (Mythreye and Blobe, 2009). Proteoglycan co-receptors (CD44, glypicans, neuropilins, syndecans, and TβRIII/betaglycan) mediate interactions with ligands, ECM proteins or other cell surface receptors to promote the formation of cell surface receptor-signaling complexes, and also to regulate cell adhesion, migration, morphogenesis, and differentiation. Among the proteoglycan co-receptors, syndecan and CD44 receptors also bind ECM molecules. Syndecan receptors bind collagens, fibronectin, and laminin and growth factors (e.g., fibroblast growth factor) to assemble signaling complexes with other receptors to control cellular differentiation and development (Yoneda and Couchman, 2003), and CD44 receptors bind to type I and IV collagens and hyaluronan to regulate cell adhesion and movement (Cichy and Pure, 2003). These ECM molecules have been exploited in the DDS not only to target cells that highly expressed those receptors in certain pathological conditions, but also to control the regulation of cellular responses.

Collagen directly interacts with four different integrin cell receptors, α_1_β_1_, α_2_β_1_, α_10_β_1_, and α_11_β_1_, depending on the type and form of collagen (Zeltz et al., 2014). α_2_β_1_ and α_11_β_1_ integrins primarily interact with the fibrillar collagen type I (e.g., α_2_β_1_ integrin mediates collagen type I binding for phagocytosis in fibroblasts (Rainero, 2016), while α_1_β_1_ and α_10_β_1_ interact with the non-fibrillar collagens IV and VI. Collagen also binds to non-integrin receptors such as discoidin domain receptors (DDR1 and DDR2), the GPVI receptor on platelets, the LAIR receptor of immune cells, the OSCAR receptor of osteoblasts, and mannose receptors (Endo180 or uPARAP) (An and Brodsky, 2016). Under particular pathological conditions, these collagen receptors are highly expressed. Endo180/uPARAP receptor is overexpressed by malignant cells in sarcomas, glioblastomas, subsets of acute myeloid leukemia (Nielsen et al., 2017). For integrins, expression of α_1_β_1_ and α_2_β_1_ was localized to scleral fibroblast focal adhesions and expression of integrin α_11_β_1_ is restricted to tumor stroma or other fibrotic disease (McBrien et al., 2006; Schnittert et al., 2018). Collagen as a ligand to target these pathological conditions thus represents a powerful therapeutic strategy.

Fibronectin binds both integrin receptors and other ECM molecules. Fibronectin type III_10_ domain which includes the RGD sequence, is the binding sites for integrins, α_5_β_1_, α_3_β_1_, α_8_β_1_, and α_v_β_3_ in a broad range of cell types and tissues (Pankov and Yamada, 2002). In particular, α_5_β_1_ integrin is required for internalization of fibronectin through caveolin-1 dependent endocytosis in myofibroblasts (Rainero, 2016). And, α_4_β_1_ and α_4_β_7_ integrins recognize the LDV and REDV motifs in the alternatively spliced V region, IDAPS in the III_14_ domain, and KLDAPT in the III_5_ domain. In addition, α_4_β_1_ and α_9_β_1_ binds the EDGIHEL sequence in the alternatively spliced EDA segment. Fibronectin also contains two heparin-binding domains within its V domain to interact with heparin and chondroitin sulfate for cell adhesion, and the fibronectin I_6__–__9_ and II_1_,_2_ domains recognize denatured collagens to clear them from blood and tissue. The expression of the various fibronectin integrin receptors depends on the pathological conditions, providing targets for DDS. The integrins α_5_β_1_ and α_v_β_3_ are upregulated in angiogenic vessels during angiogenesis (Ruoslahti, 2002); in particular, the integrin α_v_β_3_ is not expressed in healthy adult animal tissue but overexpressed during angiogenesis in tumor tissues, allowing for the targeting of integrin α_v_β_3_ with fibronectin-based, chemotherapeutic DDS.

Moreover, laminin binds various integrins receptors (α_1_β_1_, α_2_β_1_, α_3_β_1_, α_6_β_1_, α_7_β_1_, α_10_β_1_, α_6_β_4_, and α_v_β_8_) (Alam et al., 2007). Laminin-1, 2, 5, 8, 10, 11 isoforms interact with integrins α_3_β_1_ and α_6_β_1_ which regulate embryonic development, epithelial regeneration, and wound healing processes, and which also internalize to endosome as well (Das et al., 2017). Laminin binding cell receptors are highly expressed in various cancer cells types. For example, integrins α_3_β_1_ and α_6_β_1_ are overexpressed in various epithelial cancers. Amongst non-integrin receptors, laminin receptor (LAM 67R) is overexpressed on human prostate cancer cells and syndecan-2 is overexpressed in various cancer cell lines and during angiogenesis (Shukla et al., 2012). Based on expression of laminin receptors in certain pathological condition, laminin or synthetic laminin mimetic peptides as ligand are utilized as ligands to target and deliver therapeutic agents.

Chondroitin sulfate interacts with cell-surface CD44 receptors. CD44 receptors are an attractive target as they are a cancer stem cell marker which is overexpressed about four- to five-fold in metastasis and cancer progression (Goebeler et al., 1996). Owing to the interaction between chondroitin sulfate and CD44 receptor, chondroitin sulfate has been utilized in DDS to target CD44 overexpressing cancer cells and promote receptor-mediated endocytosis. The polysaccharide hyaluronic acid binds toll-like receptors, CD44, and RHAMM on cell membrane. Interactions with toll-like receptors regulate signaling in inflammatory cells and other cell types, and those with CD44 control leukocyte homing and recruitment. In addition, hyaluronic acid interactions with CD44 and RHAMM regulates tumor growth and metastasis. CD44 expression is characteristic in cells under certain pathological conditions such as infarcted myocardium, infiltrating leukocytes, wound myofibroblasts, vascular cells, and many tumor cells.

### Receptor-Mediated Endocytosis

The efficacy, biomedical function, biodistribution, and toxicity of drugs with intracellular targets of action are dictated by their internalization into the cells through interaction with the exterior of the plasma membrane and their endocytic pathway (Sahay et al., 2010; Foroozandeh and Aziz, 2018). Endocytosis occurs via two primary routes – phagocytosis and pinocytosis (Yameen et al., 2014), with phagocytosis characteristic of dendritic cells, neutrophils, monocytes and macrophages (Aderem and Underhill, 1999) and pinocytosis, which occurs via clathrin-mediated endocytosis, caveolae-mediated endocytosis, clathrin/caveolae-independent endocytosis, and micropinocytosis (Sahay et al., 2010; Yameen et al., 2014), possible for all cell types. Micropinocytosis is an actin-driven endocytic process that initiates the activation of receptor tyrosine kinases (e.g., via growth factors) to polymerize actin and form macropinosomes for cell entry. Unlike micropinocytosis, receptor-mediated endocytosis (e.g., clathrin-mediated endocytosis, caveolae-mediated endocytosis, and clathrin/caveolase-independent endocytosis) is regulated by specific interactions between a receptor and an extracellular ligand or particle (Yameen et al., 2014). Physical properties of the extracellular cargo, including particle size, shape, and surface charge, all influence the cellular uptake pathway. In addition to these physical properties, very specific ligand-receptor interactions dictate the receptor-mediated endocytosis pathways of ligand-decorated cargo.

The majority of DDS are internalized into cells through the clathrin-mediated endocytosis pathway using interactions with numerous receptors on cell membrane including transferrin, asialoglycoprotein receptor, epidermal growth factor receptor, chemokine receptors, and cell adhesion receptors (Tsuji et al., 2013; Xu et al., 2013; D’Souza and Devarajan, 2015; Phuc and Taniguchi, 2017; Hu et al., 2018; Nieto Gutierrez and McDonald, 2018). In this process, particular ligands in the extracellular fluid bind to the receptors on the surface of the cell membrane, which is rich in clathrin, to form a ligand-receptor complex (Munsell et al., 2016) that forms a clathrin-coated pit and results in the formation of clathrin-coated vesicles approximately 10 to 200 nm in diameter for internalization. After internalization, the clathrin coat on the vesicles is expelled and recycled to the plasma membrane and the vesicle fuses with the early endosomes. The cargo within early endosomes will reach lysosomes and eventually be degraded by the acidic pH and digestive enzyme of the lysosome. Given the relatively large number of binding molecules, clathrin-mediated endocytosis is a primary uptake pathway for most polymeric DDS.

Polymer-mediated nucleic acid delivery systems have been reported with both clathrin-mediated endocytosis and caveolae-mediated endocytosis as their uptake pathways, depending on the size, types, and surface charge of their cargos, and cellular microenvironment (2D vs. 3D) (El-Sayed and Harashima, 2013; Truong et al., 2019). However, trafficking of cargo through caveolae-mediated endocytosis routes enhances gene expression owing to the low or non-acidifying pathway (Rejman et al., 2005; Reilly et al., 2012). Caveolae-mediated endocytosis occurs via association of the delivery vehicle with cholesterol-rich lipid rafts in the plasma membrane for cellular entry (Sahay et al., 2010). Once cargo molecules bind to the caveolae surface rich in glycosphingolpids including GM-1 and Gb3, the caveolae engulf the cargo to form vesicles approximately 50 nm in diameter. The detached caveolar vesicles can fuse with early endosomes, but because the caveolar vesicles have neutral pH, they generally avoid fusion with lysosomes thus preventing lyososomal degradation of drug cargo.

Clathrin-and caveolae-independent endocytosis occurs without binding of the cargo to clathrin or caveolae (Yameen et al., 2014); the pathway depends instead on cell-surface molecules such as Arf-6, flotillin, Cdc42, and RhoA, involving different subtypes of internalization routes depending on the specific cell-surface molecule. Once cargo is internalized, it is usually delivered to the early endosome and trafficked though lysosomal pathways.

The ECM is constantly remodeled, via balancing of synthesis, deposition, and degradation to control tissue homeostasis, and during this process ECM molecules themselves are internalized through receptor-mediated endocytic pathways. Degradation of the ECM occurs largely through two pathways; extracellular degradation mediated by matrix metalloproteases (MMPs) and lysosomal degradation after receptor-mediated internalization (Rainero, 2016). The internalization of the most abundant component of ECM, collagen, is controlled by integrin-mediated phagocytic uptake and Endo-180 dependent clathrin mediated pathway. Fibrillar collagen type I binds to α_2_β_1_ integrin receptor, promoting internalization of collagen to early endosomes (Arora et al., 2013). On the other hand, soluble collagen type I, IV and V fragments bind Endo180 or uPARAP to internalize to endosome via the clathrin-dependent endocytic pathway (Madsen et al., 2011).

Similar to collagens, fibronectin is degraded by lysosomal degradation after endocytosis. Endocytosis of both soluble and matrix fibronectin is mediated by α_5_β_1_ integrin receptor via caveolin-1 dependent uptake (Shi and Sottile, 2008). Fibronectin binding to α_2_β_1_ integrin receptor, ultimately leading to endosomal sorting and transport to the lysosome (Lobert et al., 2010). The internalization of the major component of basement membrane, laminin, is controlled by α_3_β_1_ integrin receptor and dystroglycan for protein turnover. Interestingly, the activation of the α_3_β_1_ integrin receptor by laminin binding results in phagocytosis of other ECM molecules as well (Coopman et al., 1996). The internalization of laminin requires dystroglycan for receptor-mediated and dynamin-dependent pathways, leading to lysosomal degradation (Leonoudakis et al., 2014). Meanwhile, degradation of hyaluronic acid is controlled by multiple events. High molecular weight hyaluronic acid is degraded to smaller fragments by the extracellular hyaluronic acid-digesting enzyme, hyaluronidase 2 (Hyal 2) (Racine and Mummert, 2012). These fragments can be endocytosed by either receptor-mediated endocytosis (10^4^ Da) or micropinocytosis (10^6^ Da), depending on the molecular weight of the fragment. Hyaluronic acid fragments binding to CD44 and lymphatic vessel endothelial-1 (LYVE-1) receptors promote the endocytosis of hyaluronic acid via the clathrin-mediated pathway. The wide range of different internalization mechanisms for ECM molecules can be exploited in DDS for the selective uptake of intracellularly active drugs.

## ECM-Targeted Delivery of Particle-Based DDS

**FIGURE 2:**
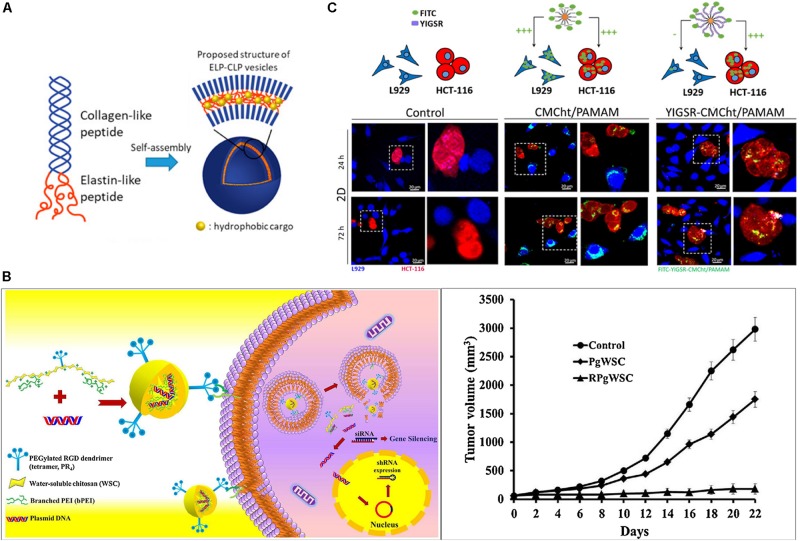
Extracellular matrix-based targeted delivery of particle-based DDS. **(A)** Schematic of ELP-CLP conjugate-based thermoresponsive nanovesicles (Luo et al., 2017). Copyright 2017. Reproduced with permission from American chemical society. **(B)** RGD dendrimer peptide modified polyethyleneimine-grafted chitosan for siRNA delivery. *In vivo* tumor growth of treatment with non-RGD-modified system (PgWSC) and RGD-modified system (RpgWSC), and non-treatment (Y.M. Kim et al., 2017). Copyright 2017. Reproduced with permission from Elsevier Inc. **(C)** Confocal images of internalization of dendrimer particles (CMCht/PAMAM and YIGSR-CMCht/PAMAM) on HCT-116 cancer cells (red) and L929 fibroblasts (blue) (Carvalho et al., 2019). Copyright Wiley-VCH Verlag GmbH. & Co. KGaA. Reproduced with permission.

Extracellular matrix molecules have been successfully formulated into particles for drug delivery applications. The chondroitin-sulfate modified CD44 receptor is able to bind to triple helical sequence from collagen Type IV (Rezler et al., 2007); Fields and co-workers thus developed CD44-binding, collagen-mimetic peptides [(GPO)_4_GVKGDKGNPGWPGAP(GPO)_4_] and used them to modify liposomes as a DDS to cancer cells with highly expressed CD44 cell receptor (Table 2). They demonstrated that doxorubicin delivered via this DDS reduced the tumor size up to 60%, compared to untreated control in a CD44^+^ mouse melanoma model (Ndinguri et al., 2012). Moreover, others have taken advantage of another collagen receptors, DDR2, which is highly expressed in fast-growing invasive tumors (Leitinger, 2014). The Brodsky group reported a recombinant collagen protein (VCLCL-DDRT) that binds DDR2 and could thus serve as a potential tumor treatment (An and Brodsky, 2016). They showed the delay of megakaryocyte migration as a result of the competition between the recombinant VCLCL-DDRT and animal collagen for binding to DDR2. In addition, our group recently has developed conjugates of the collagen-like peptide [(GPO)_4_GFOGER(GPO)_4_GG, CLP] and elastin-like peptide [(VPGFG)_6_, ELP] to serve as thermoresponsive vesicles as a drug carrier (Figure 2A) (Luo et al., 2017). This CLP-decorated vesicle has both thermally responsive assembly behavior owing to the temperature-responsiveness of the CLP domain’s triple helix formation, and a strong affinity to native collagen through collagen triple helix hybridization, and is therefore able to sequester, for at least 21 days, a hydrophobic model compound (fluorescein) in collagen type II films, with subsequent thermally triggered release. The vesicles also show high cytocompatibility with both fibroblasts and chondrocytes and essentially no activation of a macrophage cell line. The ELP-CLP conjugates have the potential to deliver intracellularly active drugs through receptor-mediated endocytosis using interactions between the GFOGER sequence on CLP and integrin receptors (α_1_β_1_, α_2_β_1_, α_10_β_1_, and α_11_β_1_) (Zeltz et al., 2014).

**TABLE 2:** Extracellular matrix protein-derived peptides as ligands to bind to cell surface receptors in drug delivery systems.

**ECM molecules**	**Peptides**	**Cell receptor**	**Application**	**References**
Collagen	GGYGGGP(GPP)_5_GFOGER(GPP)_5_GPC	α_2_β_1_	Local protein delivery	Shekaran et al., 2014
	(GPO)_4_GVKGDKGNPGWPGAP(GPO)_4_	Chondroitin sulfate modified CD44	Anti-cancer drug delivery	Ndinguri et al., 2012
	VCLCL-DDRT (Recombinant protein)	DDRs	Block the activity of cancer cell	An and Brodsky, 2016
ECM proteins	cRGD4C	α_v_β_3_ and α_v_β_5_	Anti-cancer drug delivery	Arap et al., 1998
	cRGDfC	α_v_β_3_ and α_v_β_5_	Anti-cancer drug delivery	Bibby et al., 2005
	cACRGDMFGCA	α_v_β_3_ and α_v_β_5_	VEGFR2-SiRNA delivery	Schiffelers et al., 2004
Laminin	RKRLQVQLSIRT	Syndecan	Anti-cancer drug delivery	Negishi and Nomizu, 2019
	DFKLFAVYIKYR-GGC (C16Y)	Integrin α_v_β_3_	Anti-cancer drug delivery	Hamano et al., 2012

The use of fibronectin-based molecules has also been employed for successful targeting and increased intracellular uptake of local DDS. The Akaike group incorporated fibronectin in a calcium phosphate co-precipitated, non-viral gene delivery system (Chowdhury and Akaike, 2006); the fibronectin coating in calcium phosphate and pDNA precipitate allowed cell-surface integrin receptor binding for internalization into cells and supported 100-fold higher levels of gene expression than without the fibronectin coating. In the past, direct conjugation of the cyclic RGD peptide, RGD4C, on the anticancer agent, doxorubicin, demonstrated better efficacy in suppressing tumor progression than doxorubicin alone, in mouse models bearing human breast carcinoma cells (Arap et al., 1998). The RGD peptide-modified DDS showed improved localization and intracellular uptake into cancer cells. The Jang group investigated the dendrimeric RGD peptides modified on co-polymer, which consists of polyethyleneimine and water soluble chitosan (RpgWSC), for an siRNA delivery system to target α_v_β_3_ integrin-overexpressing tumor cells for cancer therapy (Figure 2B; Kim et al., 2017). The delivery systems allow the cellular uptake of siRNA to PC3 cancer cells through microtubule-dependent micropinocytosis and clathrin-mediated endocytosis. The delivery of siRNA, via the use of their DDS with RGD (RpgWSC), for silencing the mRNA encoding the hBCL2 protein in a PC3 tumor xenograft mouse model, presented greater inhibition of tumor growth through the blocking of BCL2 protein expression, compared to a non-RGD modified delivery system (PgWSC) (Figure 2B). These results are a recent illustration of the power of employing RGD in DDS for improving delivery of intracellularly active cancer therapeutics into α_v_β_3_ integrin overexpressing tumor cells.

The active sequence peptides from laminin are able to interact with cell surface receptors, integrins, syndecans, α-dystroglycan, and CD44, to perform various biological activities like those mediated by full-length laminin. The laminin-derived RKRLQVQLSIRT (AG73) peptide was modified with PEGylated liposomes to deliver plasmid DNA in human embryonic kidney carcinoma cells, which overexpress syndecan-2 (Negishi et al., 2010; Negishi and Nomizu, 2019). On the other hand, cancer cells, including bile duct carcinoma, colorectal carcinoma, cervical cancer, and breast carcinoma, highly express the 67 KDa laminin receptor (67LR), for which the laminin-derived YIGSR sequence has high affinity. YIGSR-modified carboxylmethychitosan/poly(amidoamine) (CMCht/PAMAM) dendrimer nanoparticles were developed to drive targeted internalization into colorectal cancer cells (HCT-116 CRC cells) (Carvalho et al., 2019) via this interaction. The YIGSR-modified CMCht/PAMAM nanoparticles were more selectively internalized by HCT-116 colorectal cancer cells than by L929 fibroblasts and non-YIGSR-modified CMCht/PAMAM nanoparticles were non-selectively internalized by both types of cells (Figure 2C). Laminin-based material modification are a promising strategy to improve the specificity of the delivery system on the laminin receptor expressed cells such as tumor.

Heparin is incorporated in DDS to target overexpressed angiogenic growth factors in tumor tissues (Shing et al., 1984). Tae groups demonstrated heparin-coated PLGA nanoparticle to accumulate in the tumor in SCC7 tumor-bearing athymic mice (Chung et al., 2010). In addition, dendronized heparin-doxorubicin conjugate-based nanoparticle developed by Gu group represented the improvement of antitumor efficacy and anti-angiogenic effects in a mouse 4T1 breast cancer tumor model, compared to free doxorubicin (She et al., 2013). On the other hand, many studies have investigated the DDS incorporating hyaluronic acid or chondroitin sulfate as a ligand to target CD44-overexpressing cancer cells. Gupta and co-workers formulated polyehtylenimine (PEI) conjugated chondroitin sulfate to form complexes with plasmid DNA (Pathak et al., 2009). Their system, administrated by intravenous injection in Ehrlich ascites tumor (EAT)-bearing mice, accumulated in tumor mass to a significantly greater extent as compared to non-chondroitin sulfate-modified PEI/pDNA complex. The attachment of hyaluronic acid on liposomes loaded with doxorubicin resulted in the selective binding of the DDS on CD44-expressing murine melanoma cells, resulting in a substantial reduction in the IC_50_ (Eliaz and Szoka, 2001). In addition, Zhang group developed ternary complex based on hyaluronic acid, dexamethasone conjugated polyethyleneimine (PEI) and plasmid DNA to enhance CD44 receptor-mediated endocytosis (Fan et al., 2013). This ternary complex improved cellular uptake and nuclear transport of DNA in melanoma tumor cells, leading to the highest transfection efficiency and suppressed the growth of tumor in mice. Hyaluronic acid has also been utilized to target CD44 receptors overexpressed in macrophages as a strategy for the treatment of inflammatory disease. Pilehvar-Soltanahmadi and co-workers reported hyaluronic acid-conjugated polylactide nanoparticles encapsulated curcumin delivered to macrophage to achieve the modulation of macrophage polarity from the pro-inflammatory M1 phenotype to the anti-inflammatory M2 phenotype (Farajzadeh et al., 2018). The modification of ECM polysaccharides accomplishes the delivery of drugs at the target sites where their receptors are highly expressed.

## Ecm-Based Hydrogel Matrices for Drug Delivery

Drug transport within a hydrogel can be controlled by manipulating its mesh size and/or its interaction with drugs using chemical strategies (Merino et al., 2015; Li and Mooney, 2016; Sood et al., 2016; Oliva et al., 2017; Dimatteo et al., 2018). Hydrogels comprise crosslinked polymer networks, and drugs smaller than the network mesh size can simply diffuse through the hydrogel, whereas drugs larger than the mesh size are entrapped in the hydrogel and released upon degradation of the network. The polymer backbone and crosslinks can be degraded by either slow hydrolysis of ester bonds or peptide bonds, by the scission of thiol-based crosslinks, or by bio-responsive mechanisms such as enzyme activity (Lutolf et al., 2003; Zustiak and Leach, 2010; Wang, 2018). The degradation of hydrogels in biomedical applications can be tuned based on the local cellular environment by incorporating crosslinks comprising peptides that are degradable by different types of matrix metalloproteinases (Patterson and Hubbell, 2010). Moreover, drug release from the hydrogel can be modulated by incorporating non-covalent or covalent drug-matrix interactions (Appel et al., 2015; Li and Mooney, 2016; Ruskowitz and DeForest, 2018; Narayanaswamy and Torchilin, 2019). Non-covalent interactions include electrostatic interactions such as heparin and heparin binding proteins (Liang and Kiick, 2014; Freudenberg et al., 2016), or hydrophobic associations such as cyclodextrin and hydrophobic drugs (Mateen and Hoare, 2014). Otherwise, covalent interactions can be designed using non-cleavable and cleavable linkages between drugs and hydrogels that are incorporated via reactions such as click chemistries (e.g., copper-free click, thiol-ene, Diels-Alder reactions, and oxime and hydrazine ligation) and photochemistries (e.g., nitrobenzyl and coumarin photocleavage reactions); these reactions also are employed for hydrogel crosslinking (Christman et al., 2011; Yigit et al., 2011; Phelps et al., 2012; Ulrich et al., 2014; Kolmel and Kool, 2017; Ruskowitz and DeForest, 2018; Palmese et al., 2019). Thus, the chemical tunability of hydrogels, particularly their mesh size, crosslinking chemistry, and drug interactions, enables fine-tuned control over drug transport through the hydrogel.

### Simple Diffusion

Extracellular matrix-based hydrogels for local drug delivery not only support cells biochemically and mechanically through cell-matrix interactions, but also release the drugs into infiltrated cells. Since the hydrogel is formed by the crosslinked polymer network, the mesh space between polymer chains allows the diffusion of liquid and small molecules (Li and Mooney, 2016). Depending on the mesh size of a hydrogel, small molecule drugs can diffuse through the hydrogel and be released from the hydrogel for delivery to the surrounding cells.

Due to its structural properties, collagen is often utilized as the matrix for local drug delivery. A type I collagen matrix on the surface of polyurethane films enhanced fibroblast attachment, proliferation, and growth (Park et al., 2000). While collagen matrices provide a physiologically inspired microenvironment to cells, collagen also can control the delivery of drugs such as small molecules, proteins, and genes via simple diffusion and/or biodegradation. Collagen matrices have been loaded with a variety of small molecules such as antibiotics for wound care, cisplatin for local cancer therapy, and anti-inflammatory reagents for tissue regeneration in ophthalmology (Zilberman and Elsner, 2008; De Souza et al., 2010; Duxfield et al., 2016). Small molecule gentamicin-eluting collagen matrix [Collatamp^®^ (Schering-Plough, Stockholm, Sweden), Sulmycin^®^-Implant (Schering-Plough, United States), and Septocoll^®^ (Biomet Merck, Germany)] have been used in the clinic as wound care products to promote both granulation tissue formation and epithelialization, and to protect tissues from potential infection (de Bruin et al., 2010; Raja, 2012; Chia et al., 2014).

**FIGURE 3:**
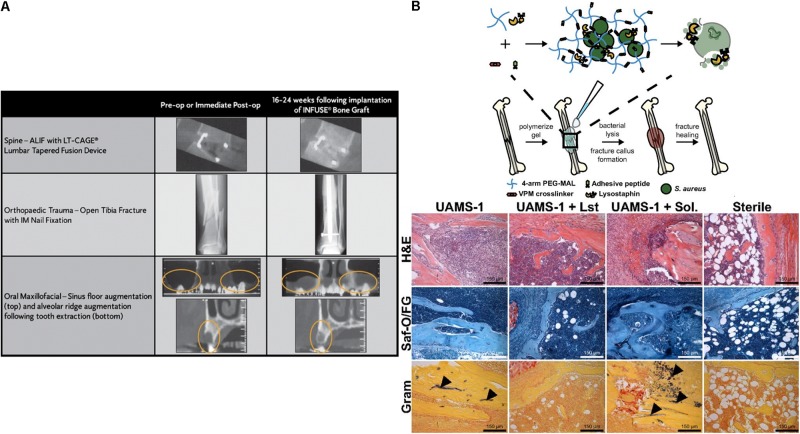
Simple diffusion of drugs from ECM based matrices. **(A)** Computed tomography (CT) images for the efficacy of INFUSE^®^ Bone Graft in clinical applications (McKay et al., 2007). Copyright 2007. Reproduced with permission from Springer Nature. **(B)** The scheme of overall study design. Histologic analysis using H&E, Saf-O/FG, and Gram staining of femurs after treating with hydrogel (UAMS-1), Lysostaphin-delivering hydrogel (UAMS-1 + Lst), and Lysostaphin, and sterilization (Johnson et al., 2018). Copyright 2018. Reproduced with permission from the National Academy of Sciences.

In addition to small molecule delivery, proteins such as growth factors can be loaded into the collagen matrix; for example the delivery of bone morphogenetic protein (BMP) from a collagen matrix has been shown to promote bone formation. Recombinant human BMP-2 (rhBMP-2)-loaded collagen matrices (INFUSE^®^ bone graft and MASTERGRAFT^®^) are available in the clinic to treat bone fracture and spinal fusion (Li and Mooney, 2016). Clinical trials using INFUSE^®^ in spinal orthopedic trauma, and oral maxillofacial applications have demonstrated the efficacy of INFUSE^®^ to form *de novo* bone (Figure 3A; McKay et al., 2007). The Garcia group created a collagen mimetic peptide (GFOGER)-modified PEG synthetic hydrogel to deliver BMP-2 to murine radial critical-sized defects (Shekaran et al., 2014). The GFOGER-modified hydrogel increased osteoprogenitor localization in the defect site and sustained release of BMP-2 to enhance bone formation and healing. In addition, the Garcia group investigated RGD and GFOGER-modified PEG synthetic hydrogels for the delivery of lysostaphin to treat *Staphylococcus aureus* infections in bone fractures (Figure 3B; Johnson et al., 2018). Based on histological analysis, lysostaphin delivery using the RGD/GFOGER-based PEG hydrogel system (UAMS-1 + Lst) demonstrated the ability of the system to reduce bacterial infection compared to the non-treatment control (UAMS-1), and these materials were shown to promote fracture repair of femoral bone in mouse such that the resulting healed tissue was similar to sterile positive control groups. A lysostaphin solution without hydrogel (UAMS-1 + Sol.) failed both in reducing bacterial infection and in improving bone repair. ECM-based hydrogel matrices create a microenvironment conductive to supporting growth of recruited cells while also controlling drug release to enhance tissue regeneration.

### ECM-Based Matrix and Drug/Carrier Interactions

Drug release from ECM-based matrix is also dependent upon drug-ECM interactions. Electrostatic and hydrophobic attractive forces between drug molecules and ECM molecules can reduce and/or prohibit drug diffusion through the network, leading to prolonged drug retention and alternate controlling parameters for release from the matrix. The electrostatic interactions between highly negative polysaccharides and drugs are employed in the sustained delivery/retention of many drugs. For example, Cool and colleagues validated the delivery efficacy of BMP-2 using thiol-modified hyaluronan (Glycosil^TM^), and these materials were compared to collagen sponges (e.g., as a mimic of INFUSE^®^ bone grafts) in terms of their influence on ectopic bone formation (Bhakta et al., 2013). The electrostatic interaction between BMP-2 and negatively charged hyaluronic acid hydrogels resulted in a low burst followed by sustained release of BMP-2, whereas collagen hydrogels showed high burst and sustained release of BMP-2. The low burst and sustained release of BMP-2 from hyaluronic acid hydrogels improved the bone formation to the greatest extent in a rat intramuscular ectopic model.

**FIGURE 4:**
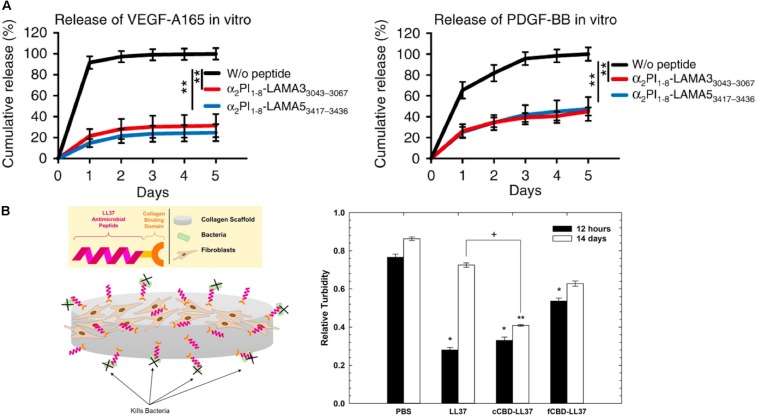
Extracellular matrix-based matrices and drug interaction-based delivery systems. **(A)** Growth factor retention in fibrin matrices with laminin-mimetic peptides (α2PI1–8-LAMA33043–3067 or α2PI1–8-LAMA53417–3436) or without peptide (**p < 0.01) (Ishihara et al., 2018). Copyright 2018. Reproduced with permission from Springer Nature. **(B)** The scheme of study design. Antimicrobial activity of LL37 and with collagen-binding domains (cCBD-LL37 or fCBD-LL37) on collagen scaffold after 12 h and 14 days (*p < 0.01, **p < 0.001, ^+^*p* < 0.05) (Lozeau et al., 2017). Copyright 2017. Reproduced with permission from Elsevier Inc.

Moreover, due to the ability of ECM molecules to interact with growth factors, ECM molecules are utilized in DDS for the sustained release of growth factors from hydrogel matrices. In particular, heparin-based hydrogels have been widely employed as growth factor carriers for tissue regeneration (Sakiyama-Elbert and Hubbell, 2000; Tanihara et al., 2001; Jeong and Panitch, 2009; Liang and Kiick, 2014; Freudenberg et al., 2016). Netti and co-workers developed porous PEG-heparin hydrogels encapsulating the angiogenic growth factor VEGF. Because of the interaction between heparin and VEGF, VEGF was released in a controlled manner and the released VEGF promoted angiogenesis *in vivo* (Oliviero et al., 2012). Also, the Werner group investigated RGD-functionalized star PEG-heparin hydrogels with a variable degree of heparin sulfation for controlled release of angiogenic growth factors from the hydrogel and capture of inflammatory chemokines in the hydrogel for the chronic wound healing applications (Freudenberg et al., 2015; Lohmann et al., 2017). In addition, the Hubbell group developed laminin-mimetic peptides, which include heparin-binding domains, and employed them to decorate a fibrin matrix for the delivery of VEGF-A165 and platelet derived growth factor PDGF-BB in a chronic wound treatment application (Ishihara et al., 2018). Since the heparin-binding domain in laminin-mimetic peptides has a strong affinity to syndecan cell surface receptors, as well as to VEGF-A165 and PDGF-BB, the system enhanced cell adhesion through interaction with syndecan, and also enabled the sustained release of growth factors from the matrix (Figure 4A). This resulted in promotion of wound healing in a type-2 diabetic mouse. With a similar approach, the Christman group applied decellularized ECM-derived hydrogels in heparin-binding growth factor delivery systems for tissue regeneration in the post-myocardial infarction (Seif-Naraghi et al., 2012). Porcine pericardia were decellularized using 1% SDS and digested with pepsin to prepare decellularized ECM-derived hydrogel with intact native sulfated glycosaminoglycans (PPM). Plasmid DNA encoding fibroblast growth factors (pFGF) in PPM injected into rats with post-myocardial infarction was still retained in the tissue after 5 days of administration, and the amount of pFGF retained was greater than the amount of bFGF retained in collagen hydrogels or in saline.

While controlled drug release via drug-ECM interactions is a powerful strategy to improve retention and sustained delivery, existing examples are mostly limited to the use of heparin-binding growth factors and charged molecules. To address this limitation, as described above, active peptide sequences from various ECM proteins have been identified and exploited in controlling the drug release from ECM-based matrices. Chemical modifications of the active sequences and their attachment to drugs or polymeric carriers enable immobilization in ECM-based hydrogel matrices for sustained drug release. Rolle and co-workers utilized a collagen-binding domain (cCBD derived from collagenase or fCBD derived from fibronectin) to tether synthetic human antimicrobial peptides, catelicidin LL37, on collagen scaffolds for treatment of wound infection (Figure 4B; Lozeau et al., 2017). Even after 14 days, LL37 with collagen domains (cCBD-LL37 and fCBD-LL37) was still retained on the collagen scaffold and showed similar levels of antimicrobial activity after 12 h. However, due to the burst release of LL37 from collagen scaffold, the antimicrobial activity of LL37-loaded collagen scaffolds was reduced at 14 days compared to 12 h. In another example, the Hubbell group developed strategies for the delivery and release of both immune checkpoint inhibitor antibodies (αCTLA4 + αPD-L1) and interleukin-2 (IL-2) using collagen-binding domains (CBDs) derived from the von Willebrand factor (vWF) A3 domain to immobilize drugs on collagen in the tumor stroma for cancer immunotherapy (Ishihara et al., 2019). Systemically administered CBD-tumor drug conjugates mainly accumulated in the tumor sites in murine cancer models, whereas non-CBD modified drugs did not. Drug delivery and release from the tumor collagen matrix-DDS interaction improved safety by eliminating antibody hepatotoxicity and by ameliorating pulmonary edema by IL-2, and it also improved efficacy through reducing the size of tumor. Overall, these examples demonstrate that the immobilization of therapeutic agents on the matrix using peptides prolongs the effectiveness of the therapeutic agents via controlled release from the scaffold.

### ECM-Based Matrix and Carrier Interaction for Intracellular Delivery

Drug delivery systems that combine these two approaches, e.g., immobilizing a drug in an ECM-based hydrogel and exploiting ECM-mediated cell uptake, have demonstrated enhanced therapeutic efficacy. In particular, this hybrid strategy will have enormous benefit on the delivery of intracellular therapeutic agents such as nucleic acids, which require DDS to facilitate cellular internalization and prevent the degradation of nucleic acids in the extra- and intracellular environments before they transfer to the appropriate cellular compartment. BMP-delivery systems using ECM-based hydrogels (INFUSE, MASTERGRAFT, OP-1) are clinically available. However, gene delivery systems often fail to meet their clinical potential due to their relative low transfection efficiency and off-target expression (Al-Dosari and Gao, 2009; Li and Mooney, 2016). The ideal gene delivery system in tissue regeneration applications should be able to sustain the delivery of active genes throughout the tissue formation process. Thus, immobilization of gene carriers in ECM-based hydrogels has the potential to achieve sustained delivery in response to cell-secreted proteases that are present during tissue repair and regeneration process, and the subsequent targeted cell uptake mediated by cell-receptor/ECM interactions.

Polymer and DNA complexes (polyplexes) have been encapsulated into scaffolds through non-specific and specific interactions between the complex and scaffold, leading to sustained DNA release from the matrix (De Laporte and Shea, 2007; Wang and Gao, 2014). Collagen-based matrix has been widely utilized to incorporate DNA complexes via non-specific interactions with the matrix to promote skin tissue repair and bone regeneration applications (Mao et al., 2009; Elangovan et al., 2014). For example, Gao and co-workers demonstrated the incorporation of cationic trimethylchitosan chloride (TMC) and DNA encoding VEGF-165 complex into the collagen-chitosan/silicone membrane bilayer dermal scaffold (TMC/pDNA-VEGF complexes loaded scaffold) to enhance angiogenesis for wound repair applications (Guo et al., 2010). Immunohistological analysis, RT-qPCR, and Western blotting analysis showed that the TMC/pDNA-VEGF complex-loaded scaffold was able to promote wound healing in incisional porcine wounds via VEGF-driven angiogenesis. The Salem group explored the delivery of polyethylenimine (PEI) and DNA encoding PDGF-B complex (Polyplex-PDGF-B) using collagen scaffolds for bone regeneration (Elangovan et al., 2014). *In vivo* studies using a calvarial defect rat model revealed that after 4 weeks of sample implantation, polyplex-PDGF-B in collagen promoted significantly higher new bone formation as compared to collagen-only scaffold, suggesting the effective approach and potential clinical translation for bone regeneration.

**FIGURE 5:**
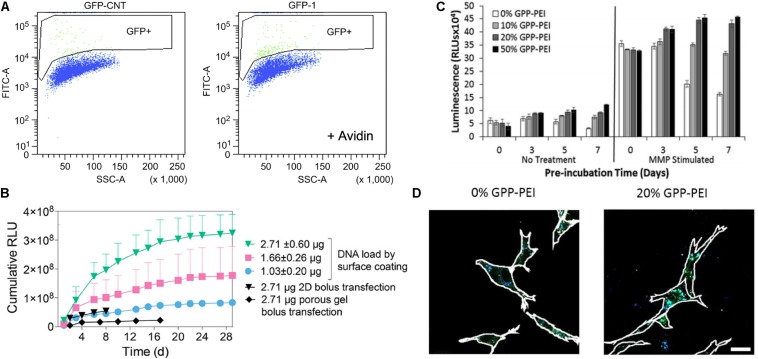
Polyplex immobilized in an ECM-based matrix for gene delivery. **(A)** Flow-activated cell sorting (FACS) analysis of biotin-functionalized pGFP polyplex immobilized in avidin-modified collagen hydrogel through avidin-biotin interaction (right graph) and avidin-free collagen hydrogel (left) (Orsi et al., 2010). Copyright 2010. Reproduced with permission from Elsevier Inc. **(B)** pGluc expression for 30 days of cell culture in the presence of immobilized pGluc polyplex on the surface of hyaluronic acid hydrogel through electrostatic interaction, and bolus transfection controls (Truong and Segura, 2018). Copyright 2018. Reproduced with permission from the American Chemical Society. **(C)** pGluc expression of immobilized GPP-PEI in the collagen hydrogel and free GPP polyplex in hydrogel after a week of pre-incubation in media with and without the presence of metalloproteinase (Urello et al., 2014). Copyright 2014. Reproduced with permission from The Royal Society of Chemistry. **(D)** Colocalization study of FITC labeled collagen (Green) with Alexa Fluor 350 labeled GPP-PEI (Blue) in NIH3T3 cells after 5 days of pre-incubation in the media (Urello et al., 2017). The scale bar is 25 μm. Copyright 2017. Reproduced with permission from Elsevier Inc.

Polyplexes also have been incorporated into the matrix via specific interactions between polyplex and matrix. Netti et al. developed gene-activated matrices through immobilization of biotin-polyethylenimine (PEI) and DNA complexes (polyplexes) in avidin-functionalized collagen matrix (Orsi et al., 2010). The immobilized polyplexes provided higher bioavailability to NIH3T3 cells recruited into the collagen matrix. The use of avidin-biotin interactions increased the transfection efficiency by approximately two-fold as compared polyplexes in collagen matrix lacking avidin-biotin linkages (Figure 5A). Moreover, Segura and co-workers recently investigated electrostatically immobilized PEI/DNA complexes (polyplexes) in porous hyaluronic acid hydrogels (Truong and Segura, 2018). The hydrogel formulation approach reduced the cytotoxicity of the polyplexes in murine mesenchymal stem cells as compared to 2D bolus transfections with multiple doses. These observations suggested that the immobilized polyplex on the hydrogel enhanced and sustained the transgene expression over 30 days of cell culture, compared to a non-coated bolus transfection (Figure 5B). In addition to these two strategies for non-covalent immobilization of poyplex to ECM hydrogels, our group has developed approaches to immobilize polyplexes in collagen hydrogels through interactions with collagen-mimetic peptides [e.g., GPP: (GPP)_3_GPRGEKGERGPR(GPP)_3_GPCCG] that have affinity for native collagen through strand invasion and triple-helical binding (Urello et al., 2014, 2016, 2017). With higher amounts of GPP incorporated in the polyplex, the polyplex was retained in the hydrogel longer, with retention up to 35 days (Urello et al., 2014). In addition, GPP-modified PEI polyplexes, after a week of pre-incubation within collagen hydrogels in media, still showed greater gene expression by murine fibroblasts compared to GPP-free polyplexes. In particular, gene transfer in MMP-stimulated cells was highly robust, suggesting potential treatment options for chronic inflammatory diseases such as chronic wounds (Figure 5C). A collagen-polyplex colocalization study revealed that the GPP-PEI, along with collagen fragments, were internalized in cells largely via caveolar endocytosis, suggesting integrin interaction with the integrin-binding sites of collagen fragments are involved in cellular internalization (Figure 5D; Urello et al., 2017). GPP-PEI and collagen hydrogel interactions allowed both the controlled release and ligand-mediated efficient endocytosis into cells.

## Summary and Future Prospects

For the past several decades, significant progress has been made in the development of targeted DDS using both local administration and ligand-based active targeting strategies. Hydrogel-based local delivery and ligand-cell interaction-mediated delivery enable drugs such as biomacromolecules (e.g., growth factors or genes) and small molecules to better localize at the target sites. Owing to the biological versatility of ECM molecules, ECM-based DDS have been applied not only to provide structural and biochemical signals to cells, but also to serve as ligands for cell receptors in specific pathological conditions to improve therapeutic efficacy of growth factor, gene, and small molecule treatments. However, despite progressive improvements, many challenges and unmet clinical needs still remain, particularly for intracellularly active drugs such as genes, which require control over cellular uptake mechanisms for optimized delivery and activity.

The innovative combination of these two targeting approaches using immobilizing drug carriers in ECM-based hydrogels has generated promising cell-responsive gene-activated matrices for regenerative medicine and functional tissue repair. ECM scaffolds not only function as substrates for cell infiltration, organization, and differentiation, but also enable resident cells to efficiently uptake genes on demand to supply essential tissue inductive factors. However, many challenges remain in further developing this type of DDS to, for example, enable the delivery of multiple drugs from a single system, or provide mechanisms for on-demand drug release with a high level of control to a specific cell type. The sequential signaling of multiple growth factors typically regulates tissue repair and regeneration. Although researchers have demonstrated the release of multiple drugs, obtaining release of a specific molecule with optimal timing remains a challenge. Further, despite the advances in targeting, materials that localize only at or in their target cells are still difficult to design due to the lack of cell-specific gene expression relevant to a given disease physiology. Use of multiple ECM-inspired peptides in conjunction may offer a promising strategy to increase affinity to a particular cell type, using information about the cell’s natural ECM receptor expression patterns, or to promote the sequential delivery of a series of drugs in a desired profile. In the future, ECM molecule-based DDS are likely to have an increasingly significant impact on disease treatment and tissue regeneration.

## Author Contributions

All authors conceived the layout, the rationale, and the plan of this manuscript. JH wrote the first draft of the manuscript that was iteratively improved by MS and KK.

## Conflict of Interest

The authors declare that the research was conducted in the absence of any commercial or financial relationships that could be construed as a potential conflict of interest.
